# Supplementary material for: Violation of the Sphericity Assumption and Its Effect on Type-I Error Rates in Repeated Measures ANOVA and Multi-Level Linear Models (MLM)
Source: Front Psychol. 2017 Oct 17;8:1841. doi: 10.3389/fpsyg.2017.01841 (PMC5651023; doi:10.3389/fpsyg.2017.01841)
Supplement: Supplementary file 1 [file DataSheet1.pdf]

Appendix

```
* IBM SPSS Syntax-example for the first run of the simulation study based on 100 participants.



* sphericity condition.


*population with correlated dependent variables.

set  RNG=MT seed=1000  MXLOOPS=1000000.
INPUT PROGRAM.
 LOOP #1 = 1 to 500000.
 DO REPEAT z = z1 to z10.
 COMPUTE z = NORMAL(1).
 END REPEAT.
 END CASE.
 END LOOP.
 END FILE.
END INPUT PROGRAM.
execute.

compute y1=z1.
compute y2=z2.
compute y3=z3.
compute y4=z4.
compute y5=z5.
compute y6=z6.
compute y7=z7.
compute y8=z8.
compute y9=z9.


* select 100 participants for the first run.
compute Pbn=$casenum.
select if (Pbn<=100).
execute.


*repeated measures ANOVA with and without corrections (Greenhouse-Geisser, Huynh-Feldt).

GLM y1 to y9
  /WSFACTOR=time 9 Polynomial
  /METHOD=SSTYPE(3)
  /PRINT=ETASQ
  /CRITERIA=ALPHA(.05)
  /WSDESIGN=time .
```

```
*MLM with compound-symmetry.

VARSTOCASES
 /MAKE sphericity_violation FROM y1 to y9
 /INDEX=Index.

MIXED sphericity_violation BY Index
 /CRITERIA=CIN(95) MXITER(100) MXSTEP(10) SCORING(1) SINGULAR(0.000000000001)
HCONVERGE(0, ABSOLUTE) LCONVERGE(0, ABSOLUTE) PCONVERGE(0.000001,
ABSOLUTE)
 /FIXED=Index | SSTYPE(3)
 /METHOD=REML
 /REPEATED=Index | SUBJECT(Pbn) COVTYPE(CS).

*MLM unstructured covariances.

MIXED sphericity_violation BY Index
 /CRITERIA=CIN(95) MXITER(100) MXSTEP(10) SCORING(1) SINGULAR(0.000000000001)
HCONVERGE(0, ABSOLUTE) LCONVERGE(0, ABSOLUTE) PCONVERGE(0.000001,
ABSOLUTE)
 /FIXED=Index | SSTYPE(3)
 /METHOD=REML
 /REPEATED=Index | SUBJECT(Pbn) COVTYPE(UN).



*population with correlated dependent variables.


* sphericity violation.

set  RNG=MT seed=1001  MXLOOPS=1000000.
INPUT PROGRAM.
 LOOP #1 = 1 to 500000.
 DO REPEAT z = z1 to z10.
 COMPUTE z = NORMAL(1).
 END REPEAT.
 END CASE.
 END LOOP.
 END FILE.
END INPUT PROGRAM.
execute.

compute y1=SQR(0.80)*z10+SQR(0.20)*z1.
compute y2=z2.
compute y3=SQR(0.80)*z10+SQR(0.20)*z3.
compute y4=z4.
compute y5=SQR(0.80)*z10+SQR(0.20)*z5.
compute y6=z6.
compute y7=SQR(0.80)*z10+SQR(0.20)*z7.
compute y8=z8.
compute y9=SQR(0.80)*z10+SQR(0.20)*z9.
```

```
* select 100 participants for the first run.
compute Pbn=$casenum.
select if (Pbn<=100).
execute.


*repeated measures ANOVA with and without corrections (Greenhouse-Geisser, Huynh-Feldt).

GLM y1 to y9
  /WSFACTOR=time 9 Polynomial
  /METHOD=SSTYPE(3)
  /PRINT=ETASQ
  /CRITERIA=ALPHA(.05)
  /WSDESIGN=time .


*MLM with compound-symmetry.

VARSTOCASES
 /MAKE sphericity_violation FROM y1 to y9
 /INDEX=Index.

MIXED sphericity_violation BY Index
 /CRITERIA=CIN(95) MXITER(100) MXSTEP(10) SCORING(1) SINGULAR(0.000000000001)
HCONVERGE(0, ABSOLUTE) LCONVERGE(0, ABSOLUTE) PCONVERGE(0.000001,
ABSOLUTE)
 /FIXED=Index | SSTYPE(3)
 /METHOD=REML
 /REPEATED=Index | SUBJECT(Pbn) COVTYPE(CS).

*MLM unstructured covariances.

MIXED sphericity_violation BY Index
 /CRITERIA=CIN(95) MXITER(100) MXSTEP(10) SCORING(1) SINGULAR(0.000000000001)
HCONVERGE(0, ABSOLUTE) LCONVERGE(0, ABSOLUTE) PCONVERGE(0.000001,
ABSOLUTE)
 /FIXED=Index | SSTYPE(3)
 /METHOD=REML
 /REPEATED=Index | SUBJECT(Pbn) COVTYPE(UN).
```
